# Metabolomics analysis of the hippocampus in a rat model of traumatic brain injury during the acute phase

**DOI:** 10.1002/brb3.1520

**Published:** 2019-11-17

**Authors:** Fei Zheng, Yan‐Tao Zhou, Dan‐Dan Feng, Peng‐Fei Li, Tao Tang, Jie‐Kun Luo, Yang Wang

**Affiliations:** ^1^ College of Electrical and Information Engineering Hunan University Changsha China; ^2^ Laboratory of Ethnopharmacology Institute of Integrated Traditional Chinese and Western Medicine Xiangya Hospital Central South University Changsha China

**Keywords:** acute traumatic brain injury, hippocampus, metabolomics, protein database analysis

## Abstract

**Background:**

Traumatic brain injury (TBI) has increased in rank among traumatic injuries worldwide. Traumatic brain injury is a serious obstacle given that its complex pathology represents a long‐term process. Recently, systems biology strategies such as metabolomics to investigate the multifactorial nature of TBI have facilitated attempts to find biomarkers and probe molecular pathways for its diagnosis and therapy.

**Methods:**

This study included a group of 20 rats with controlled cortical impact and a group of 20 sham rats. We utilized mNSS tests to investigate neurological metabolic impairments on day 1 and day 3. Furthermore, we applied metabolomics and bioinformatics to determine the metabolic perturbation caused by TBI during the acute period in the hippocampus tissue of controlled cortical impact (CCI) rats. Notably, TBI–protein–metabolite subnetworks identified from a database were assessed for associations between metabolites and TBI by the dysregulation of related enzymes and transporters.

**Results:**

Our results identified 7 and 8 biomarkers on day 1 and day 3, respectively. Additionally, related pathway disorders showed effects on arginine and proline metabolism as well as taurine and hypotaurine metabolism on day 3 in acute TBI. Furthermore, according to metabolite–protein database searches, 25 metabolite–protein pairs were established as causally associated with TBI. Further, bioinformation indicated that these TBI‐associated proteins mainly take part in 5′‐nucleotidase activity and carboxylic acid transmembrane transport. In addition, interweaved networks were constructed to show that the development of TBI might be affected by metabolite‐related proteins and their protein pathways.

**Conclusion:**

The overall results show that acute TBI is susceptible to metabolic disorders, and the joint metabolite–protein network analysis provides a favorable prediction of TBI pathogenesis mechanisms in the brain. The signatures in the hippocampus might be promising for the development of biomarkers and pathways relevant to acute TBI and could further guide testable predictions of the underlying mechanism of TBI.

## Graphical abstract

Metabolomics was used to reveal the gain a metabolic perturbation of TBI in rats. Acute TBI was susceptible to metabolic disorders, and joint metabolite‐protein network analysis provides a favorable prediction to TBI pathogenesis mechanism in the brain.

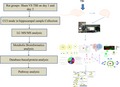

## 1 INTRODUCTION

Traumatic brain injury (TBI) is described as a leading cause of accidental death and disability worldwide, annually affecting an estimated 2.8 million cases in the USA and with an annual economic impact of more than $60 billion (CDC, 2013; Taylor, Bell, Breiding, & Xu, 2017). Given its multifunctional and heterogeneous processes, there is a paucity of studies of the complex and dynamic pathophysiology of TBI, resulting in slow progress and no better strategies for TBI diagnosis and treatment. Therefore, it is urgent to elucidate the underlying pathological mechanisms of TBI, particularly during the acute phase, which will be beneficial for properly treating TBI.

Appropriate diagnosis and prognosis of abnormalities by candidate circulating biomarkers have been shown to aid in predicting treatment of diseases (Roberts & Gerszten, 2013; Zhang, Sun, & Wang, 2012). Omics has grown considerably in terms of applications and contributions to integrative analysis as a global nontargeted approach to systems biology research (Chen et al., 2012; Cho, Labow, Reinhardt, van Oostrum, & Peitsch, 2006; Cisek, Krochmal, Klein, & Mischak, 2016; Hillmer, 2015). In this global nontargeted profiling, the perturbed biological issues are typically profiled and studied in term of circulating biomarkers and specific pathways (Mones et al., 2011; Hyungwon & Norman, 2011). Additionally, metabolism plays a powerful role for a gaining holistic understanding of various biological matrices by globally measuring small‐molecule metabolite levels (Beale et al., 2018; Zampieri & Sauer, 2017). Thus, metabolomics offers a potential strategy for the development and improvement of a method to decipher the biological processes of TBI as well as to elucidate its pathological mechanisms.

In previous literature, some studies on TBI have focused on metabolic disturbances in a mammalian model after brain injury by performing a high‐throughput profiling metabolomic investigation. Evidence found that TBI is a consequence of multiple mechanisms, mainly including oxidative stress, excitotoxic damage, membrane disruption, neuronal injury, energy failure, and mitochondrial disorder (Borgens & Liu‐Snyder, 2012; Coles et al., 2004; Lakshmanan et al., 2010; Mendes Arent, de Souza, Walz, & Dafre, 2014; Menon et al., 2004; Rossi, Oshima, & Attwell, 2000; Timofeev et al., 2011). Likewise, the studies showed that traumatic abnormalities resulted in the development of synaptic plasticity impairments and cognitive deficits in the hippocampus due to neuroinflammation (Cortese & Burger, 2017; Jaworski et al., 2011; Szarka et al., 2019). Furthermore, initial primary injuries that occur in the hippocampus due to mechanical trauma are followed by secondary injury processes around its surrounding organizations (Lynch, 2004; Whitlock, Heynen, Shuler, & Bear, 2006). Additionally, previous studies found that altered concentrations of metabolites in the hippocampus might be related to energy metabolism dysregulation and excitatory neurotransmission at later stages of traumatic injury by untargeted H‐NMR metabolomics (McGuire et al., 2019; Viant, Lyeth, Miller, & Berman, 2005).

Traumatic brain injury is a severe cranial injury induced by sudden external force. Once TBI occurs, acute phase‐based brain may suddenly experience concussion, contusion, bleeding, edema, inflammation, oxidative stress, and neurological dysfunction (Yang et al., 2019). Generally, we take 1–3 days after onset of TBI as time points to explore the effects of experimental acute TBI (Bentz et al., 2010; Yang et al., 2019; Zhang et al., 2014). Even though some studies associated with brain metabolism in the hippocampus were established, only a few metabolic biomarkers were studied by a paucity of studies exploring the pathophysiology of its acute TBI.

**Figure 1:**
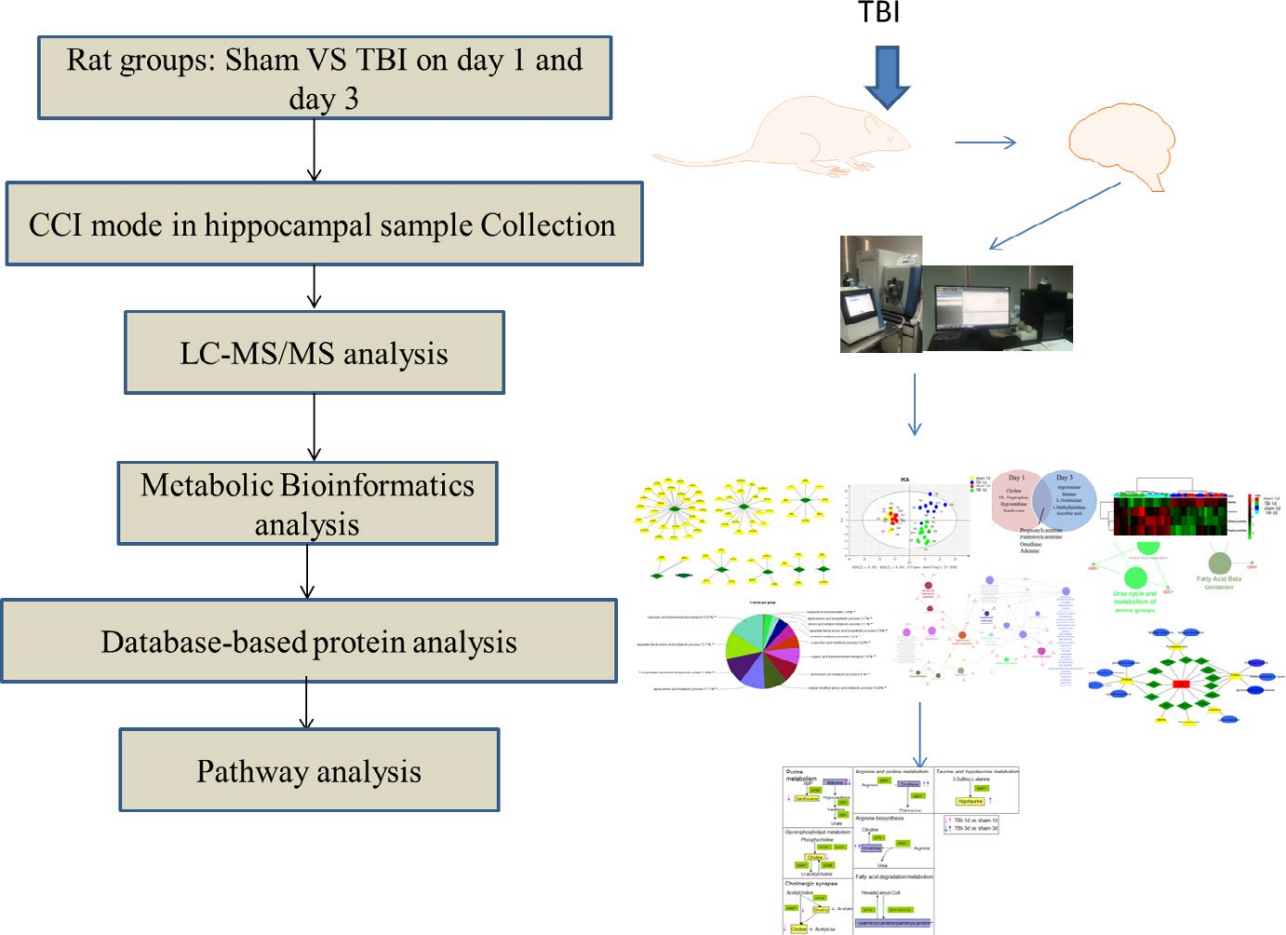
Flowchart of the design and analysis in this study

Here, we investigated the application of lipid chromatography–mass spectrometry analyses (LC‐MS/MS) based metabolomics to an acute TBI rat brain model and observed the profound metabolic changes in the hippocampus through high‐throughput analysis. The resulting metabolic fingerprints revealed the significant metabolites in response to acute trauma and characterized the affected pathways involved in the metabolic mechanism accompanying brain injury. Moreover, analysis of protein–metabolite subnetworks revealed proteins that potentially participate in the development of acute TBI, as elucidated from an evaluation of the metabolite–protein database. Additionally, according to the TBI‐protein subnetworks searched in the database, we may infer associations between metabolites and TBI that were affected by the dysregulation of related enzymes and transporters. This inference approach for identifying novel associations might provide new insights to achieve a better understanding of pathological events in the brain after acute TBI. These alterations could uncover favorable indicators for the early detection and assessment of prognosis outcome for TBI treatment. The workflow in this study is presented in Figure 1.

## 2 MATERIAL AND METHODS

### 2.1 Ethical approval

All study procedures were approved by the Animal Care and Use Committee of Xiangya Hospital of Central South University. All animal experimental protocols were conducted in accordance with relevant laboratory guidelines established by Central South University.

### 2.2 Animals preparation and Controlled Cortical Impact (CCI) model sampling

We purchased forty male Sprague–Dawley (*SD*) rats weighing 200–250 g from the Laboratory Animal Research Center of Central South University. Experimental rats were on a daily schedule for at least 1 week and then housed for 12 hr to freely eat food and water before tests. Rats were randomly divided into two groups: (a) the Sham group (*n* = 20) and (b) the controlled cortical impact group (CCI) (*n* = 20); each injury group was randomly divided into two subgroups consisting of day 1 and day 3 postinjury. A TBI model was employed as previously described [27]. First, the animals underwent intraperitoneal anesthesia with 3% of pentobarbital (50 mg/kg). Next, a 5 mm craniotomy was performed with a dental drill over the right portion of the skull. The sham animals underwent the same craniotomy but without any impact to the brain. The CCI injury was performed by an automated controlled pneumatic impact device (Precision Systems & Instrumentation, PSI TBI‐0310 Impactor, Fairfax Station, VA). The parameters of the damage indexes consisted of a 5 mm deep contusion, impact velocity of 6.0 m/s, and dwell time of 500 msec (Xing et al., 2016; Zhou et al., 2017). Thereafter, the incision was closed routinely with interrupted 3–0 silk sutures, and the animal was placed into an electric blanket to maintain its body temperature for 45 min. Then, the animals were returned to clean cages.

The animals were administered injected 5% of pentobarbital on 1 and 3 days after TBI. The rats were then infused with 200 ml of cold 0.9% normal saline for perfusion. The hippocampus tissues of the injured side were collected, froze immediately in liquid nitrogen, and stored in a −80°C freezer until analysis was performed.

### 2.3 Modified neurological severity score (mNSS) assessment

To evaluate neurological function, we used mNSS tests to investigate the neurological impairments (Xing et al., 2016). Briefly, the eighteen points of mNSS include are derived as follows: motor (6 points), sensory (2 points), beam balance (6 points), reflexes absent, and abnormal movements (4 points). One point is given for failure of one task, and no points are given for success. Animals that have higher scores are expected to have more severe neurological impairment (normal score: 0, maximal deficit score: 18). After CCI, mNSS was tested on the first day and the third days.

### 2.4 LC‐MS/MS analysis

The brain tissues were homogenized in H_2_O. The protein assay was performed on each of the individual homogenates. For metabolites, 100 µl of the homogenates were extracted. The extraction was carried out using MeOH: acetonitrile (ACN) (1:1, v/v). Afterward, the samples were vortexed for 30 s and sonicated for 10 min. To precipitate proteins, the samples were incubated for 1 hr at −20°C, followed by centrifugation for 15 min at 20,000 *g* and 4°C. The supernatant was removed and evaporated to dryness in a vacuum concentrator. The dry extracts were then reconstituted in ACN, vortexed for 30 s, and sonicated for 10 min. To remove insoluble debris, the extracts were centrifuged for 15. Finally, the supernatants were transferred to HPLC vials and stored at −80°C prior to LC/MS analysis.

Samples were separated on an amide column. And the mobile phase A consists of 25 mM ammonium acetate‐mixed water and 25 mM Ammonium hydroxide and mobile phase B ACN use 4 μl injection volume with a 0.4 ml/min flow rate. High‐performance liquid chromatography (HPLC) was used to determinate metabolism with the generic HPLC gradient listed (time: 0.0 min; 1.0 min; 11.0 min; 14.0 min; 16.5 min; 18.5 min; 20.5 min; 25.0 min; 25.1 min; and 34.0 min. A: 10%; 10%; 13%; 20%; 30%; 50%; 80%; 80%; 10%; 10%. B: 90%; 90%; 87%; 80%; 70%; 50%; 20%; 20%; 90%; and 90%). The HPLC system was the autosampler. The HPLC chromatography analysis was carried out on an Agilent 1,260 Infinity HPLC (Agilent J & W Scientific, Folsom, CA, USA) equipped with a Waters amide column (2.1 × 100 mm, 3.5 μm). The average width of each chromatographic peak was 20 s. The total cycle time was adjusted according to the average width of each chromatographic peak.

MS/MS analysis coupled with HPLC was carried out on a Q‐Exactive MS/MS (Thermo) in both positive and negative ion modes in this study. The relevant MS conditions for the probe were as follows: auxiliary gas was 13; sheath gas was 40; aux gas heater temperature was 400°C; capillary temperature was 350°C; S‐lens was 55; and spray voltage was 3.5 kV for positive mode and negative mode. DDA method utilized as the following conditions: full scan range was 60–900 (m/z); normalized collision energies were 10, 17, 25 or 30, 40, 50; automatic gain control MS1 was 3e6 and ddMS2 was 2e5; isolation window was 1.6 m/z; resolution MS1 was 70,000 and ddMS2 was 17,500; maximum injection time MS1 was 100 ms and ddMS2 was 45 ms. The full scan method was performed as follows: the full scan range was 60–900 (m/z); the automatic gain control was 3e6 ions; resolution was 140,000; and the maximum injection time was 100 ms.

### 2.5 Data analysis and statistics

After obtaining the exact mass of each feature, we identified the metabolites by ChemSpider (http://www.chemspider.com/) and mzCloud (https://www.mzcloud.org/) which are the Mass Spectral Databases. For metabolomics analysis, SIMCA‐P software (version 11.0; Umetrics AB, Umea, Sweden) was used for multivariate statistical analysis (PCA, PLS‐DA, OPLS‐DA) with log10 transformation and Pareto scaling on each statistical platform. A principal component analysis (PCA) model was visualized for the clustering trend based on the metabolites from samples. The results were visualized in term of score plots, in which each point represented an individual sample. Partial least squares‐discriminant analysis (PLS‐DA) and orthogonal projections to latent structures discriminant analysis (OPLS‐DA) models were employed for supervised class discrimination among groups according to recommended parameters. The values of R2X and R2Y were estimated to explain the model fitness, and Q2 was described for the predictive accuracy of its class model. Reliability validation of the PLS‐DA model was subsequently tested by a 100 times permutation. In the OPLS‐DA analysis, significant differential metabolites were selected based on their variable importance with projection (VIP) values >1 and a false discovery rate (FDR) <0.05. To identify the potential affected biochemical pathways, all these differential identifications on day 1 and day 3 were investigated by MetaboAnalyst 3.0 (http://www.metaboanalyst.ca/) based on the availability of Kyoto Encyclopedia of Genes and Genomes (KEGG). Specifically, metabolite–protein networks and TBI‐related protein networks were searched from the online Human Metabolome Database (HMDB) and GeneCards databases to visualize the association between them. The biological pathway analysis, biological process, and correlation networks were constructed based on the KEGG database in cytoscape (version 3.5) software and its package Gingo. Student's *t* test was used for statistical analysis with *p* values <.05 designated as significant. Data are presented in the form of the mean ± *SEM*.

## 3 RESULTS

### 3.1 Neurological deficits assessments on day 1 and day 3

**Figure 2:**
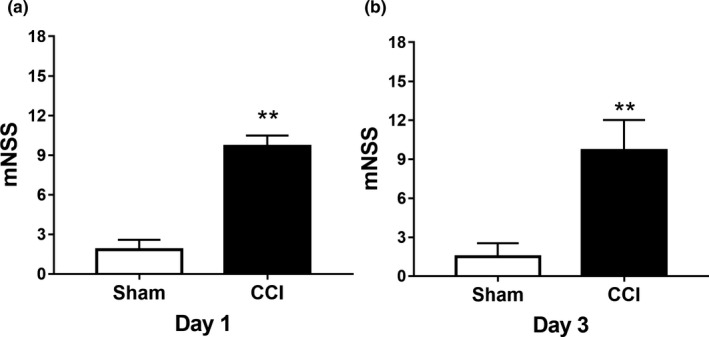
TBI effect on neurological impairments of hippocampal tissue on day 1 and day 3. (a) On day 1, the mNSS of the TBI group was significantly different from that of the sham group, which indicated the successful establishment of CCI models. (b) On day 3, the mNSS of the TBI group was significantly different from that of the sham group, which indicated the successful establishment of CCI models. **p < .01. mNSS, modified neurological severity score

According to the results of mNSS, we found that the mNSS scores in the Sham group were less than 3 points. Compared with the Sham group, CCI significantly induced neurological impairments both on day 1 and day 3 (*p* < .01) (Figure 2).

### 3.2 Global metabolic fingerprints changed on day 1 and day 3

**Figure 3:**
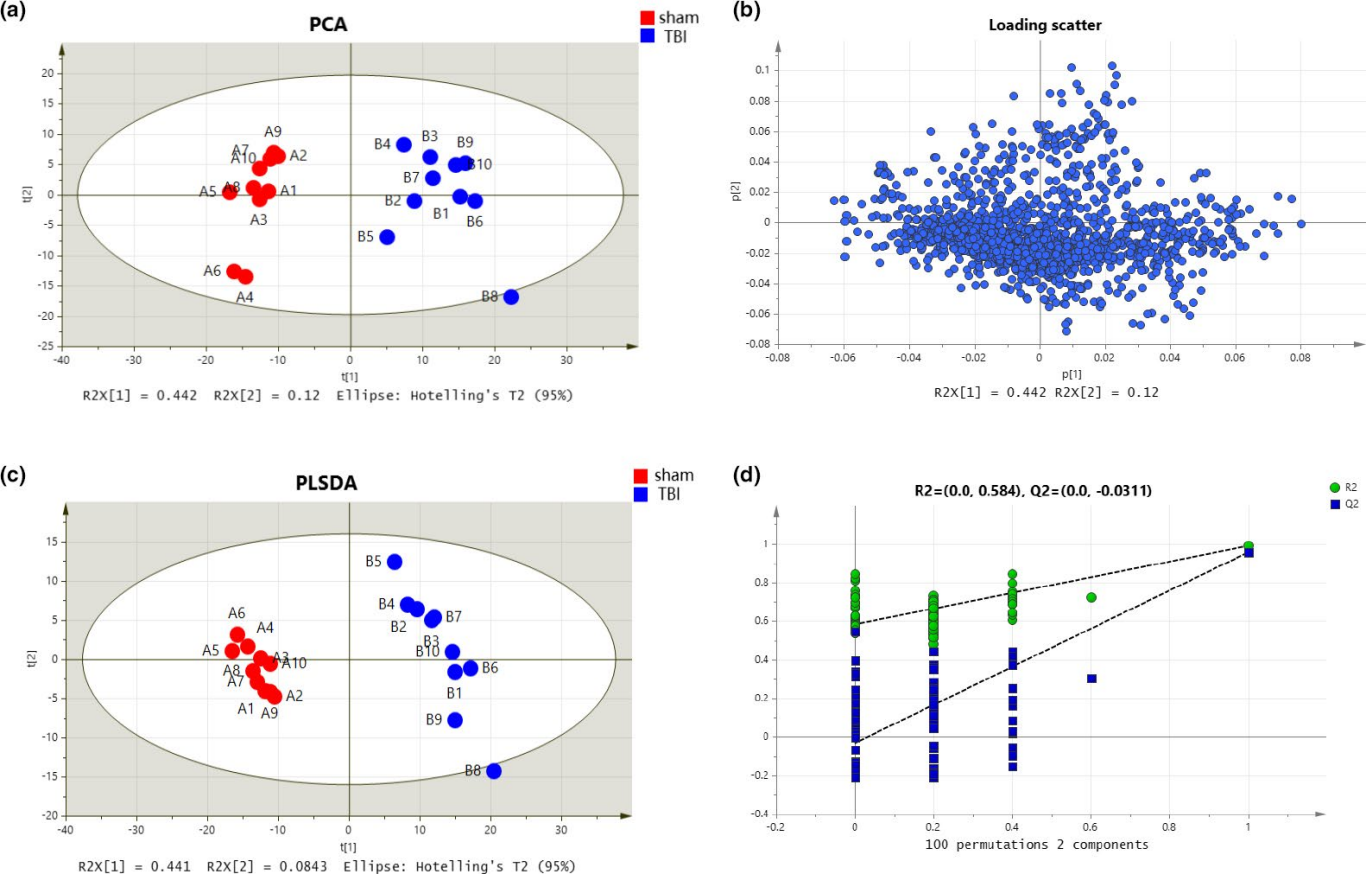
Metabolic profiles of hippocampal samples distinguish the TBI group and sham group on day 1 (red for sham group and blue for TBI group). (a) Loading plot of the metabolic profiling for the PCA model. (b) The corresponding PCA score plots comparing the sham and TBI groups. (c) Score plot of metabolic profiling for the PLS‐DA model. (d) Validation plots of the OPLS‐DA model of the TBI group and sham group were obtained through 100 permutation tests

**Figure 4:**
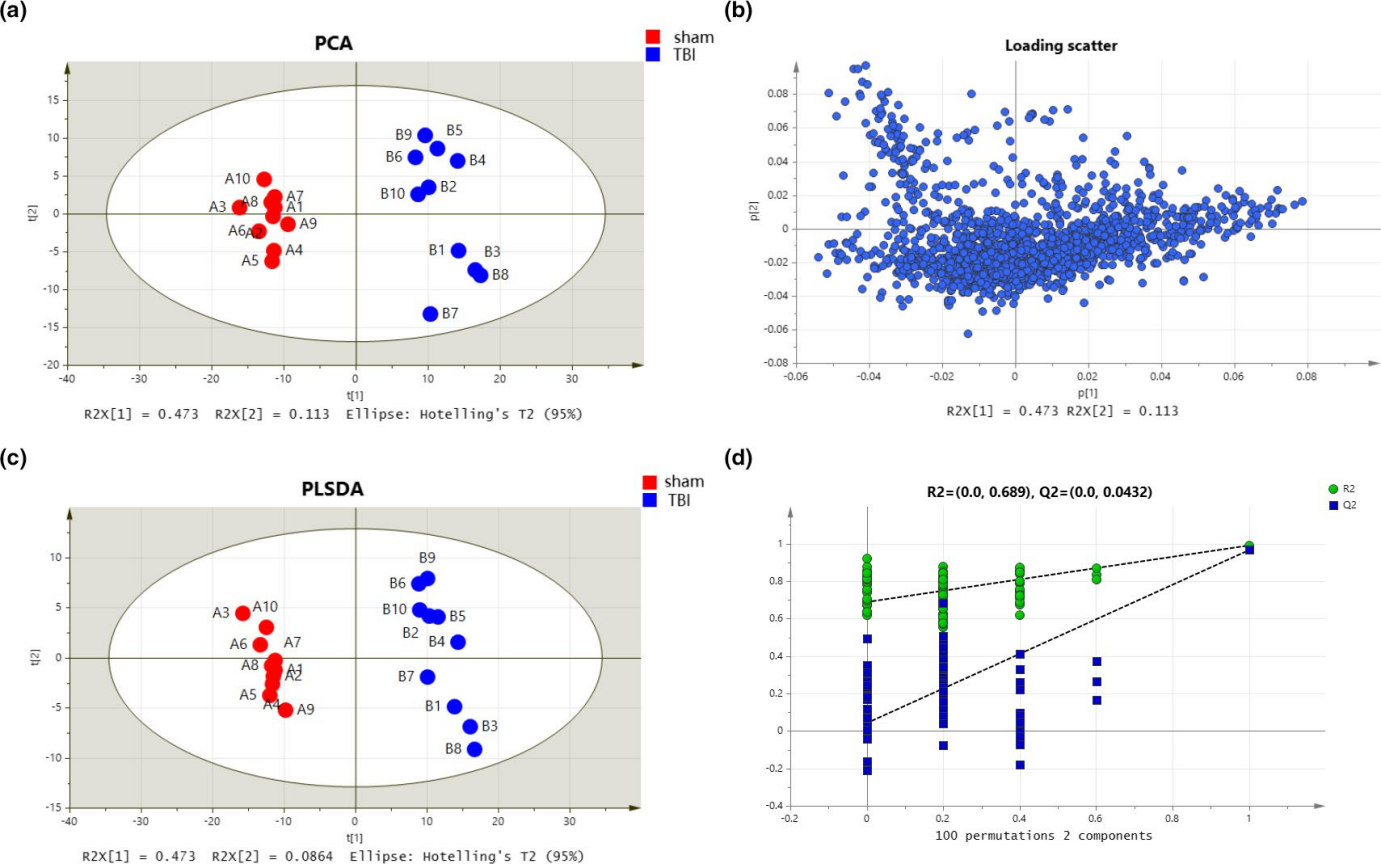
Metabolic profiles of hippocampal samples distinguish the TBI group and sham group on day 3 (red for sham group and blue for TBI group). (a) Loading plot of the metabolic profiling for the PCA model. (b) The corresponding PCA scores plots comparing the sham and TBI groups. (c) Score plot of metabolic profiling for the PLS‐DA model. (d) Validation plots of the OPLS‐DA model of the TBI group and sham group were obtained through 100 permutation tests

**Figure 5:**
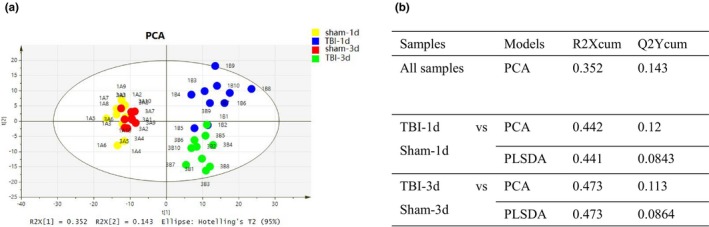
The ability of metabolic profiling to distinguish the TBI and sham groups on day 1 and day 3. (a) Score plot of PCA of metabolic profiling in TBI controls (yellow for sham on day 1, blue for TBI on day 1, red for sham on day 3, and blue for TBI on day 3). (b) Table for R2X (cum) and Q2Y (cum) parameters in PCA and PLS‐DA models on day 1 and day 3

Detailed metabolite fingerprints were obtained by employing LC‐MS/MS analysis of hippocampal samples at 24 hr and 72 hr. Multiple pattern recognition models such as PCA, PSL‐DA, and OPLS‐DA were adopted to better visualize the subtle similarities and differences among sample set. First, PCAs of the 1725 hippocampal metabolites revealed significant separation of the TBI group and blank control group during hippocampus metabolism considering the footprints at 24 hr and 72 hr, respectively (Figures 3a and 4a). The corresponding PCA loading plots identified TBI‐induced metabolic changes in the hippocampus region of the brain by comparing the sham and TBI groups on day 1 and day 3, respectively (Figures 3b and 4b). Figures 1c and 3c demonstrated a clear distinction between the TBI group and sham group on day 1 (R[1] = 0.441, R[2] = 0.084) and on day 3 (R[1] = 0.473, R[2] = 0.086). Meanwhile, 100 times random permutation tests were established to show a good quality model without overfitting with R2 (green circles) and Q2 (dark blue boxes) values as the permuted analysis (bottom left) that were lower than the corresponding original R2 and Q2 values (top right) on day 1 and day 3, respectively (Figures 3d and 4d). The summary results of the PLS‐DA analysis showed that the metabolic profiles of TBI rats were statistically distinguishable from non‐TBI rats in hippocampus tissue on day 1 and day 3, which can be appreciated from Figure 5b. In Figure 5a, the PCA of sham versus TBI together showed the separation of the TBI and sham groups, as well as that of their separation, took place from day 1 to day 3.

### 3.3 Metabolic biomarkers were significantly different on day 1 and day 3

**Figure 6:**
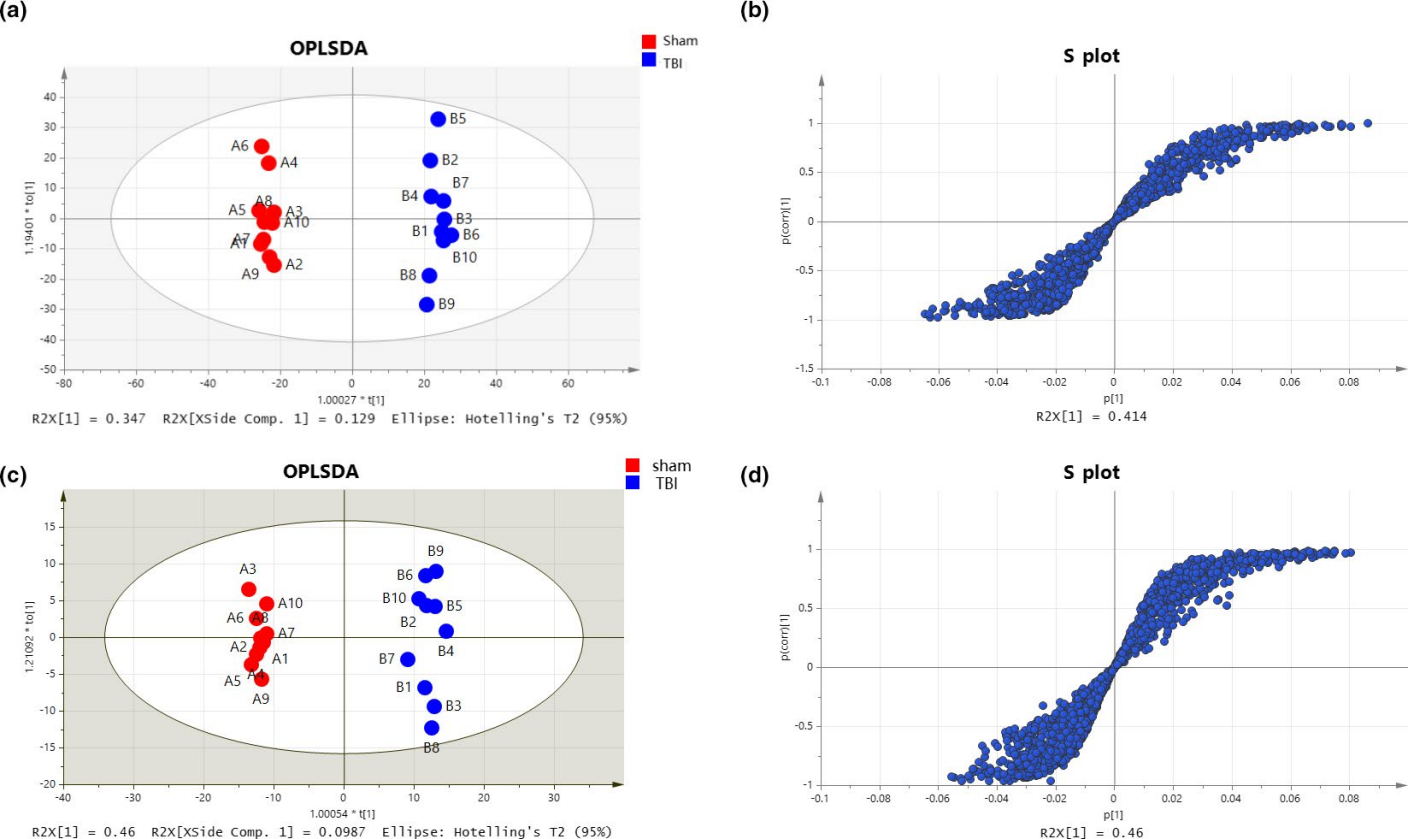
Metabolic profiles with OPLS‐DA of hippocampal samples distinguishing the TBI group and sham group. (a) Metabolites in the acute TBI and sham rat groups in the OPLS‐DA model on day 1. (b) S plot used in our biomarker selection on day 1. (c) Metabolites in the acute TBI and sham rat groups in the OPLS‐DA model on day 3. (d) S plot used in our biomarker selection on day 3

**Figure 7:**
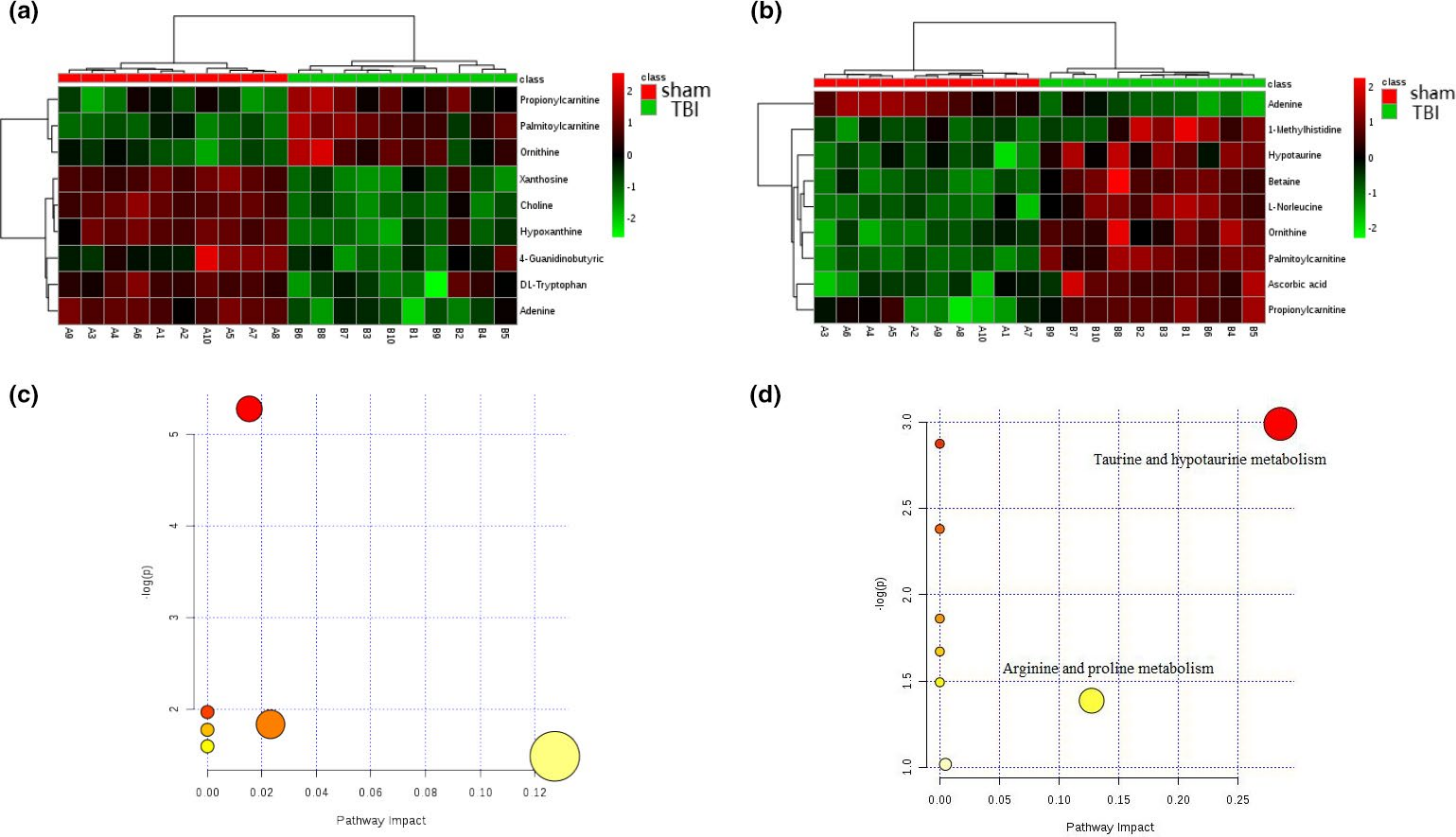
Metabolic profiles with metabolite compositions and metabolic pathways. (a) Heat map analysis for metabolite compositions between TBI and sham groups on day 1. (b) Heat map analysis of metabolite composition of the TBI and sham group on day 3. (c) The metabolic pathways from MetaboAnalyst 3.0 on day 1. (d) The metabolic pathways from MetaboAnalyst 3.0 on day 3. Two enriched pathway names have been added

**Figure 8:**
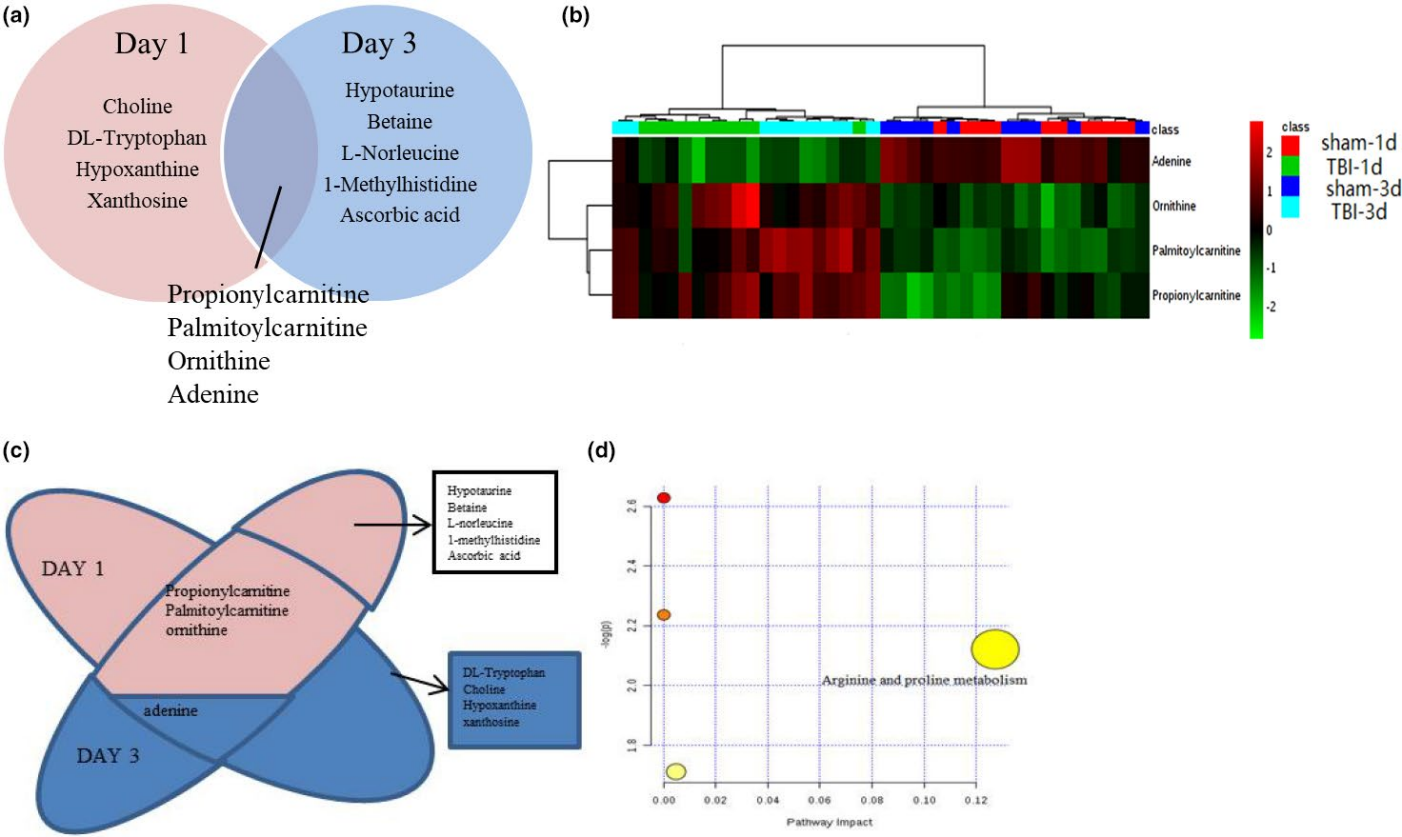
Metabolic profiles with differential metabolite compositions and metabolic pathways. (a) The Venn diagram shows the differentially regulated metabolites identified on day 1 and day 3. The metabolites in the cross section of the Venn diagram are the differentially regulated metabolites on day 1 and day 3. (b) Heat map analysis of metabolite composition changes between the TBI and sham groups. (c) The Venn diagram shows the differentially regulated metabolites identified on day 1 and day 3, with red for upregulated and blue for downregulated. (d) The metabolic pathways from MetaboAnalyst 3.0 for metabolite composition changes on day 1 and day 3. One enriched pathway name has been added

Next, a supervised OPLS‐DA model was adopted for the separated footprint of the metabolic model (Y2R = 0.414 and Q2 = 0.108) on day 1 (Figure 4a) and (Y2R = 0.46 and Q2 = 0.099) on day 3 (Figure 6b). Additionally, VIPs in each compound were obtained in an S plot diagram on day 1 and day 3 (Figures 6b and 6c). We screened 8 metabolites employed with VIP > 1.0, *p* < .05, and FC > 1.5 as significantly different biomarkers for day 1 (Table 1) and 9 for day 3 (Table 2). Among the 8 differential compounds, 3 compounds were upregulated and 5 were downregulated in the acute phase on day 1 after TBI. Among 9 compounds, 1 was downregulated and 8 were upregulated on day 3. These results indicated that the disturbance of VIP metabolites surged as time passed from day 1 to day 3 after TBI, as visualized in heat maps (Figure 7a,b). Afterward, 2 pathways (taurine and hypotaurine metabolism and arginine and proline metabolism) were significantly identified by MetaboAnalyst software by using its “pathway analysis” model on day 3 (Figure 7d). As shown in Figure 8a,b, the 4 common different metabolites (propionylcarnitine, palmitoylcarnitine, ornithine, and adenine) on both day 1 and day 3 revealed the same trend of change in the hippocampus of TBI, as shown in the Venn diagram and the heat map. A Venn diagram in Figure 8c was constructed to show the characteristic summary of different metabolites on day 1 and day 3 (red means upregulated, and blue means downregulated). Moreover, through analysis with MetaboAnalyst, arginine and proline metabolism was significantly enriched by the 4 common different metabolites both on day 1 and day 3 (Figure 8d).

**Table 1:** Differential metabolites on day 1 TBI

Name	MW	RT [min]	KEGGID	VIP^a^	*p* value^b^	FDR^c^	FDR^d^	FC
DL‐Tryptophan	204.09	6.62	C00525	1.01	1.00E−04	3.38E−03	3.38E−03	0.56
Propionylcarnitine	217.13	8.268	C03017	1.10	1.38E−03	4.36E−03	4.36E−03	2.06
Palmitoylcarnitine	399.33	2.015	C02990	1.14	8.62E−05	4.75E−04	4.75E−04	2.14
Ornithine	132.09	21.979	C00077	1.18	5.26E−03	1.31E−02	1.31E−02	2.59
Choline	103.10	7.12	C00114	1.30	1.88E−09	8.10E−07	8.10E−07	0.44
Adenine	135.05	1.37	C00147	1.37	1.08E−07	5.31E−06	5.25E−06	0.38
4‐Guanidinobutyric acid	145.09	17.1	C01035	1.42	1.48E−01	2.23E−01	2.23E−01	0.11
Hypoxanthine	136.04	2.295	C00262	1.52	4.03E−07	1.16E−05	1.16E−05	0.32
Xanthosine	284.08	3.284	C01762	1.83	1.25E−05	1.17E−04	1.17E−04	0.20

a Variable importance in the projection (VIP) based on OPLS‐DA models.

b *p* values were calculated by using a *t* test.

c False discovery rate (FDR) was identified as <0.01.

d Fold change (FC). Metabolites with FC > 1.5 or FC < 1 were identified.

**Table 2:** Differential metabolites on day 3 TBI

Name	MW	RT [min]	KEGGID	VIP^a^	*p* value^b^	FDR^c^	FDR^d^	FC
Hypotaurine	109.02	13.65	C00519	1.07	1.45E−04	6.59E−04	6.59E−04	1.79
Ornithine	132.09	21.98	C00077	1.08	1.13E−04	5.35E−03	5.34E−04	1.74
Betaine	117.08	6.17	C00719	1.08	4.87E−05	2.94E−04	2.94E−04	1.70
l‐Norleucine	131.09	6.07	C01933	1.10	3.44E−06	4.60E−05	4.60E−05	1.72
1‐Methylhistidine	169.08	19.36	C01152	1.13	1.03E−02	2.15E−02	2.15E−02	2.55
Ascorbic acid	176.03	1.75	C00072	1.17	1.90E−04	8.19E−04	8.19E−04	1.99
Propionylcarnitine	217.13	8.27	C03017	1.28	6.29E−05	3.52E−04	3.52E−04	2.17
Palmitoylcarnitine	399.33	2.01	C02990	1.57	3.37E−06	4.58E−05	4.55E−05	2.87
Adenine	135.05	2.14	C00147	1.58	6.00E−05	3.44E−04	3.44E−04	0.32
4‐Guanidinobutyric acid	145.09	17.1	C01035	1.11	1.26E−01	1.81E−01	1.81E−01	0.23

a Variable importance in the projection (VIP) based on OPLS‐DA models.

b *p* values were calculated by using a *t* test.

c False discovery rate (FDR) was identified as <0.01.

d Fold change (FC). Metabolites with FC > 1.5 or FC < 1 were identified.

### 3.4 Metabolite–protein network analysis

**Figure 9:**
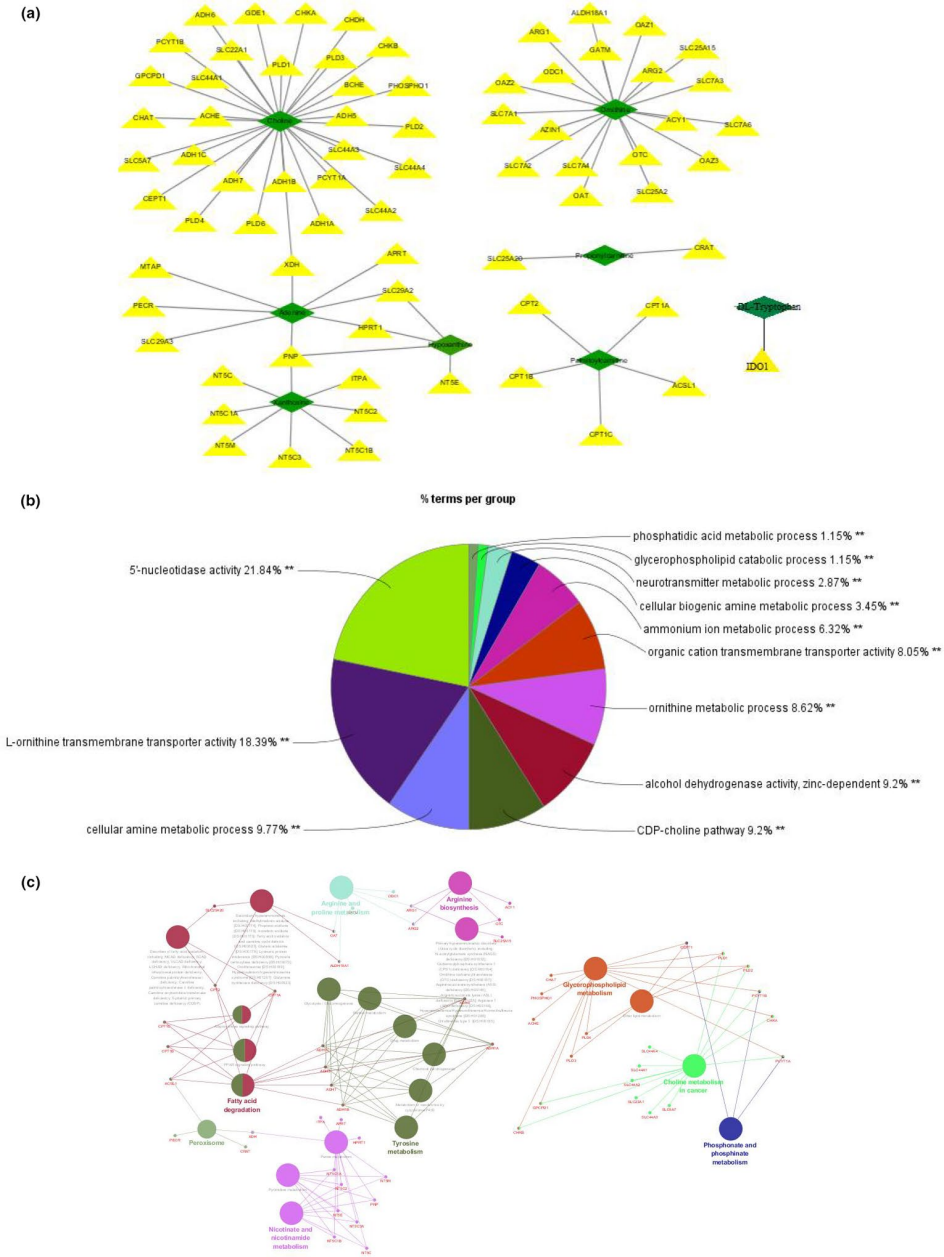
GO biological process analysis and molecular function annotation of metabolite‐related proteins. (a) Metabolite–protein network on day 1. (b) GO biological process analysis of metabolite‐related proteins. (c) Pathways distribution and diseases related to the proteins

**Figure 10:**
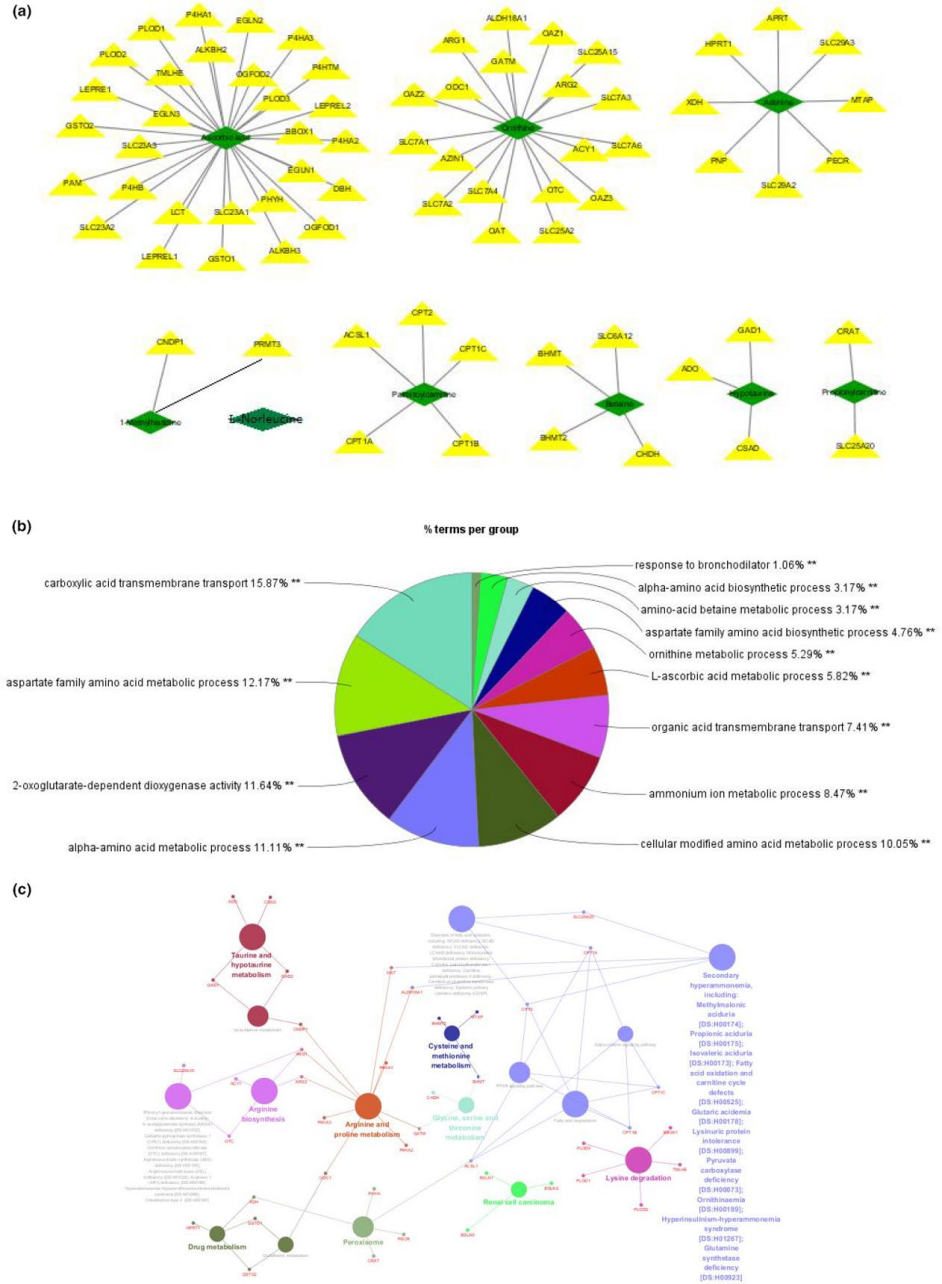
GO biological process analysis and molecular function annotation of metabolite‐related proteins. (a) Metabolite–protein network on day 3. (b) GO biological process analysis of metabolite‐related proteins. (c) Pathway distribution and related diseases of proteins

To determine the dependability of the potential targets of TBI in the acute period, a metabolite–protein network was established based on databases shown in Figures 9a and 10a. A total of 117 proteins, 111 enzymes, and 6 transporters (SLC44A1, SLC5A7, SLC22A1, SLC44A4, SLC44A2, and SLC44A3) relevant to the 13 potential targets were searched in the HMDB database (Tables 3 and 4). The 117 proteins were imported to cytoscape for analysis of those that are involved in 5′‐nucleotidase activity and carboxylic acid transmembrane transport based on the database. According to the pie chart in Figure 9b, there were 12 biological processes involving 75 related proteins on day 1, with l‐ornithine transmembrane transporter activity showing the highest proportion followed by 5′‐nucleotidase activity. According to Figure 10b, there were 13 biological processes involving 42 related proteins on day 3, with aspartate family amino acid metabolic processes showing the highest proportions followed by carboxylic acid transmembrane transport. Moreover, we constructed the protein–pathway networks according to the KEGG databases to gain a systematic view of the relationships among protein, pathway, and disease associations by using cytoscape (Figures 9c and 10c). The results are summarized and demonstrate that 65 related proteins are involved in 9 main pathways, including tyrosine metabolism, fatty acid degradation, and nicotinate and nicotinamide metabolism at the top three on day 1. Furthermore, there are 9 main pathways, the top three of which being drug metabolism, taurine and hypotaurine metabolism, and arginine biosynthesis, with 73 related proteins involved on day 3. In addition, the results from the cytoscape analysis showed that the same 2 disease associations (urea cycle disorder and disorder of fatty acid oxidation) were addressed both on day 1 and day 3 according to the association of 117 proteins.

**Table 3:** Metabolite‐related proteins on day 1 TBI

Name	KEGGID	HMDB ID	Court of proteins
DL‐Tryptophan	C00525	HMDB0013609	1
Propionylcarnitine	C03017	HMDB0000824	2
Palmitoylcarnitine	C02990	HMDB0000222	5
Ornithine	C00077	HMDB0000214	19
Choline	C00114	HMDB0000097	30
Adenine	C00147	HMDB0000034	8
Hypoxanthine	C00262	HMDB0000157	4
Xanthosine	C01762	HMDB0000299	9

**Table 4:** Metabolite‐related proteins on day 3 TBI

Name	KEGGID	HMDB ID	Court of proteins
Hypotaurine	C00519	HMDB0000965	4
Ornithine	C00077	HMDB0000214	19
Betaine	C00719	HMDB0000043	4
l‐Norleucine	C01933	HMDB0001645	‐
1‐Methylhistidine	C01152	HMDB0000001	2
Ascorbic acid	C00072	HMDB0000044	29
Propionylcarnitine	C03017	HMDB0000824	2
Palmitoylcarnitine	C02990	HMDB0000222	5
Adenine	C00147	HMDB0000034	8

### 3.5 TBI‐protein network analysis

**Figure 11:**
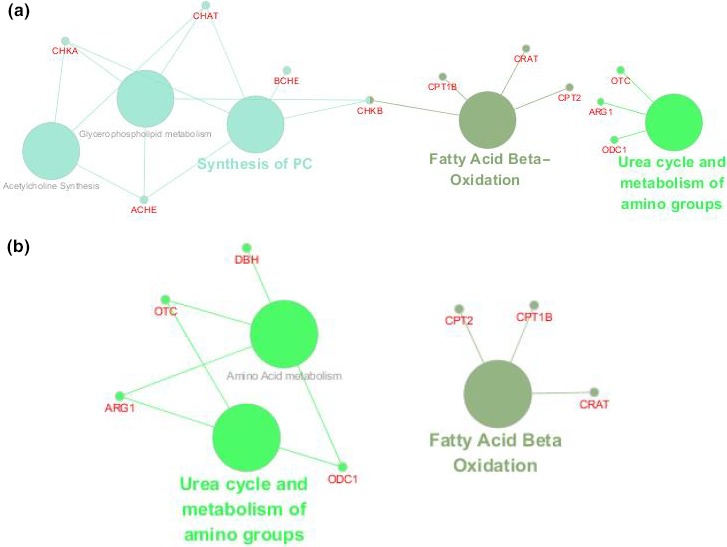
Protein‐pathway network view in cytoscape. (a) Twelve TBI‐related proteins and their enriched pathways on day 1. (b) 7 TBI‐related proteins and their enriched pathways on day 3

**Figure 12:**
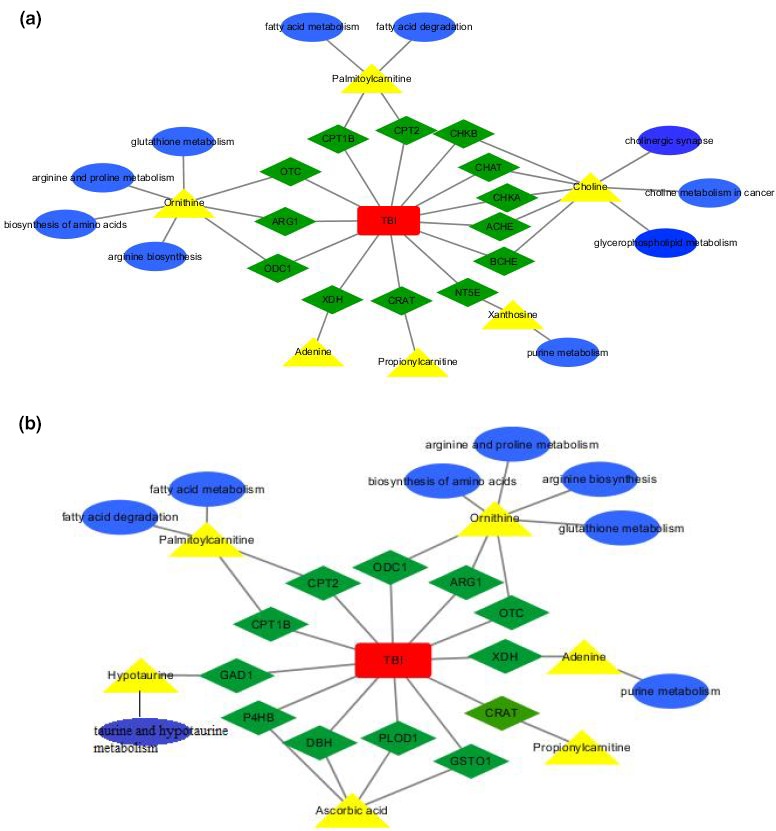
TBI–protein–metabolite–pathway network view. (a) TBI–protein–metabolite–pathway network view on day 1 based on the KEGG database. (b) TBI–protein–metabolite–pathway network view on day 3 based on the KEGG database

**Figure 13:**
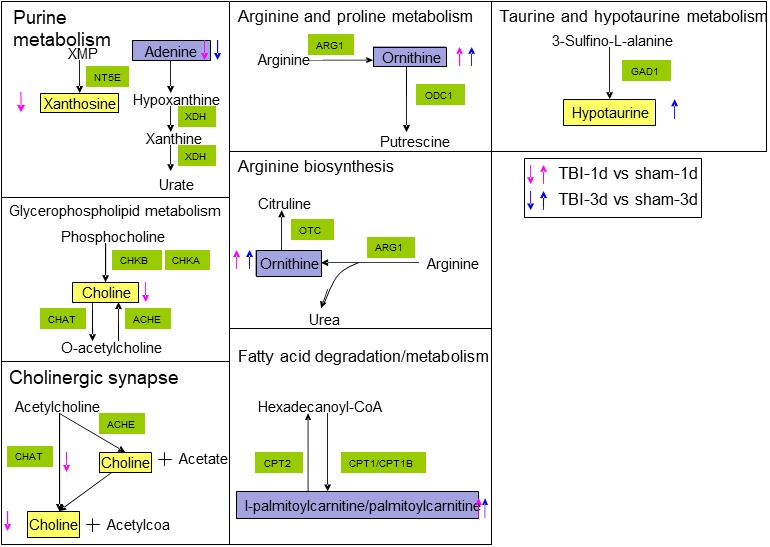
Disturbed metabolites are shown by yellow rectangles. Up arrows show upregulation trends. Down arrows show downregulation. Red arrows represent metabolites that changed on day 1. Blue arrows represent metabolites that changed on day 3. The metabolites indicated with purple color showed changes on both day 1 and day 3

To explore the underlying metabolic mechanisms in TBI, we used the GeneCards database to obtain TBI‐related proteins from among the 117 metabolite‐related proteins shown in Figure 11 with green diamonds. Furthermore, from the cytoscape analysis, we constructed networks with pathways (blue ellipse) associated with TBI (red rectangle) and disease‐related proteins (green diamond). Based on the KEGG database and PubMed, TBI‐protein associated networks were identified, which are shown with different color for each compound in Figure 11a,b for day 1 and day 3, respectively. From this integrative analysis, we may determine the main role of metabolites (yellow triangle) in TBI disease through the effects of related proteins in purine metabolism, fatty acid degradation, glycerophospholipid metabolism, and arginine and proline metabolism on day 1 (Figure 12a). Arginine and proline metabolism, taurine and hypotaurine metabolism, and glycerophospholipid metabolism may be mainly affected by metabolite‐related proteins during the acute process of TBI on day 3 (Figure 12b). The representative network analysis in Figure 12 shows that 9 differential metabolites and 13 related proteins were eventually identified, which may contribute to the dysregulation of metabolic pathways, including purine metabolism, glycerophospholipid metabolism, taurine and hypotaurine metabolism, cholinergic synapse, arginine and proline metabolism, fatty acid metabolism, and arginine biosynthesis. In Figure 13, notably, 3 metabolites (adenine, ornithine, and palmitoylcarnitine) had the same trend from day 1 to day 3. These results might reveal the function of proteins as metabolites when contributing to TBI in the acute phase.

## 4 DISCUSSION

This study performed perturbation profiling in hippocampus tissue of TBI rats at the acute phase using metabolism based on LC‐MS/MS coupled with bioinformation analysis. The metabolomic profiling analysis indicated an abnormal metabolic pattern of the hippocampus in acute CCI rats on day 1 and day 3, composed of 13 patient signatures and 2 metabolic pathways. Furthermore, a total of 151 metabolite‐related proteins were observed based on the HDMB database, including 25 related to TBI that were found in via the GeneCards database. Our proteomics database analysis led to the identification of 24 main biological processes and the same 2 TBI‐related pathways: taurine and hypotaurine metabolism and arginine biosynthesis. In summary, this untargeted metabolomic assessment of TBI‐associated brain tissue demonstrated unique and intricate alterations in hippocampal metabolites, as well as tracked continuous and progressive changes in multiple metabolic pathways. Our findings provide new insight and improved understanding of the physiological alterations resulting from acute TBI within the hippocampus at the protein and metabolite levels and may help to more accurately predict potential therapeutic outcomes of TBI.

The hippocampus is an important structure in the brain that is involved in multiple functional systems, including cognitive functions and working memory (Menon, 2016). Additionally, the hippocampus is an area of interest in TBI because investigations of its damage in TBI might improve the understanding of the neurobiological pathophysiology of TBI impairment (Bae et al., 2019; Campos‐Pires et al., 2019). Therefore, investigation of hippocampal metabolite alterations and their modulations associated with at the appropriate time windows of acute TBI could facilitate novel therapeutic strategies in the treatment of brain trauma. Our results from this metabolomic study indicate that acute TBI disease‐specific patterns can be identified in the alteration profiles through the acute stages of TBI development. Thus, 13 significant different metabolites (DL‐tryptophan, propionylcarnitine, palmitoylcarnitine, ornithine, choline, adenine, hypoxanthine, xanthosine, betaine, l‐norleucine, 1‐methylhistidine, hypotaurine, and ascorbic acid) were selected as potential candidate biomarkers in the acute phase. Additionally, the KEGG analysis indicated that there were 2 amino acid metabolism pathways enriched by these metabolites in the hippocampus of acute TBI, including taurine and hypotaurine metabolism and arginine and proline metabolism. These results provide evidence that an acute TBI‐dependent hippocampal study based on the metabolome could be developed to predict dynamic metabolic alterations in TBI pathophysiology.

Changes in the levels of TBI injury‐related metabolites in our study are notable: The level of ornithine changed significantly in this study. Ornithine has been previously shown to decrease polyamine inflammation and influence TBI energy metabolism and its secondary pathologies following severe TBI; this metabolite has been used in patients as a therapeutic approach during the first 24 hr after TBI (Jeter, Hergenroeder, Ward, Moore, & Dash, 2012; Zahedi et al., 2010). Another compound that increased in relation to acute TBI in this study is ascorbic acid, which was related to the level of the cerebral antioxidant defenses in TBI‐induced rats over 6 hr (Tavazzi et al., 2005; Watson, 1993). These results may suggest consistency with studies of cascades leading to inflammation and excitotoxicity at the early stages of hippocampal TBI.

Of note, there were three metabolites (DL‐tryptophan, l‐norleucine, and 1‐methylhistidine) in our study that have not been shown in previous studies of TBI. Furthermore, to our knowledge, this is the first time that we found decreases in xanthosine, hypoxanthine, and adenine and increases in palmitoylcarnitine and hypotaurine in the hippocampus of TBI during the acute phase. Xanthosine was reported as a product of increased oxidative stress involving purine catabolism (Yao et al., 2010). Additionally, findings regarding palmitoylcarnitine suggested an association between changes in its levels and beta‐oxidation disorders and energy consumption (Leonardi, Rock, Jackowski, & Zhang, 2007; Perevoshchikova, Quinlan, Orr, Gerencser, & Brand, 2013). Hypoxanthine was found to broadly affect purines with respect to neuron induction of glutamate excitotoxicity (Jackson et al., 2017). Adenine originally accompanied myocardial ATP decreases during periods of anoxia or ischemia (Frenguelli, 2019; Coffman, Lewis, & Gregg, 1960). Hypotaurine is highlighted as an abundant amino acid in body and is related to the antioxidant response with taurine (Bibekananda, Mahesh, & Anil, 2017; Stipanuk, Simmons, Karplus, & Dominy, 2011). Accordingly, these considerable changes in this study suggested that physiological and pathological processes are involved in oxidative stress; specifically, excitotoxicity with glutamate, fatty acid beta‐oxidation, energy requirements, and the inflammatory response may occur during the acute period in the hippocampus of TBI. These links between causes from trauma and effects on pathology could inform the treatment strategies after acute TBI.

Two metabolites (propionylcarnitine and choline) in our study have been observed in the hippocampal region associated with TBI in the chronic phase. Propionylcarnitine was associated with ATP and glutathione, neurons, oxidative stress, and energy consumption, as previously reported in the hippocampal region in the subacute period after ischemia (Al‐Majed et al., 2006; Paulson, Traxler, Schmidt, Noonan, & Shug, 1986). Choline supply was found to be a key neurotransmitter in multiple brain functions and a key element of neuronal membranes as a formation substrate (Abreu‐Villaca, Filgueiras, & Manhaes, 2011; Albright, Tsai, Friedrich, Mar, & Zeisel, 1999; Obeid, 2013; Zeisel & Niculescu, 2006). Specifically, (Meck, Smith, & Williams, 1989) showed that choline intake affected various brain function indexes in offspring including memory and spatial cognition. Notably, changes in these two metabolites were found in the hippocampal region associated with TBI at the acute phase in this study. Therefore, the altered levels of propionylcarnitine and choline offer evidence for the pathophysiology of TBI involved in biological processes such as oxidative stress, energy depletion, and inflammation from the acute to the chronic phase in the brain. On the other hand, the overlap of metabolites (propionylcarnitine, palmitoylcarnitine, ornithine, and adenine) found on both day 1 and day 3 in the hippocampus might indicate that oxidative stress disease progression involves palmitoylcarnitine, propionylcarnitine, and adenine, as well as ornithine inflammation, which continuously acts in the TBI hippocampus during the acute period.

In addition, our study showed significant pathological profiles of arginine and proline metabolism as well as taurine and hypotaurine metabolism in the hippocampus of acute TBI. In arginine and proline metabolism, arginine as a precursor produced ornithine as a substrate, ultimately producing nitric oxide or various metabolites (Castillo, Beaumier, Ajami, & Young, 1996; Li et al., 2001). Additionally, ornithine was associated with cognitive impairment and was also found in plasma metabolomics profiles in acute TBI rats (Fei et al., 2017; Wu et al., 2014). In the case of taurine and hypotaurine metabolism, the pathway enrichment analysis suggested its dysregulation in Alzheimer's disease and the development of perturbed markers in Parkinson's disease (Graham et al., 2018; Gui, Liu, Zhang, Lv, & Hu, 2015). Conversely, the results from (Menzie, Prentice, & Wu, 2013) and (Wen et al., 2018) highlighted that taurine and hypotaurine metabolism can protect neurons against inflammatory response or may be related to hypoxic preconditioning. These results may provide evidence that brain diseases, including TBI, can be attributed to the dysregulation of taurine and hypotaurine metabolism as well as arginine and proline metabolism. Thus, these significantly enriched pathways have potential utility as suitable diagnostic and therapeutic targets in brain tissue against TBI.

Deep insight into molecular mechanisms of TBI in terms of potential downstream targets and their interactions were investigated by a metabolomics approach followed by proteomics database information analysis. Our proteomics database analysis showed an accumulation of 25 different proteins upon TBI which influenced 12 pathways that were identified based on these proteins. In particular, our proteomics database analysis showed that taurine and hypotaurine metabolism and arginine and proline metabolism were in agreement with our metabolome results in the hippocampus during the acute phase. Of these pathways, ODC1, ARG1, and GAD1 were involved in 2 novel critical pathways and interacted with their related metabolites in this study. Ornithine decarboxylase (ODC) could convert ornithine to putrescine and induce polyamine biosynthesis at limiting rates in several central nervous system (CNS) injuries (Longo et al., 1993; van Steeg et al., 1989). ARG1 was reported to regulate neurodegenerative disorders in immunity for the central nervous system in chronic TBI compared with a sham group (Andreasson et al., 2016; Sonia, David, & Mark, 2017; Tagge et al., 2018). GAD1 was associated with an increase in posttraumatic seizure (PTS) risk due to excitotoxicity associated with gamma‐amino butyric acid (GABA) transmission after acute TBI (Darrah et al., 2013). Taken together, the cross talk between metabolites and their related proteins leading to the accumulation of deep molecular mechanism of TBI is suggested. Thus, the metabolite‐related protein compounds identified in the response to TBI strongly support the findings of metabolite involvement in hippocampal TBI at the acute phase in this study. Additionally, further cross‐talk pathway analysis between metabolites and proteins level is expected to be investigated to determine pathophysiological influences on TBI.

We have attempted to demonstrate a metabolome profile response to a TBI disease environment in the acute phase, but several limitations should be recognized in our study. First, we only used LC‐MS/MS‐based technology to detect TBI brain tissue metabolomics. Additional metabolomics platforms such as gas chromatography–mass spectrometry (GC–MS) and nuclear magnetic resonance (NMR) should be required to verify our findings. Moreover, our present study was performed with a relatively small sample size. Other studies with larger sample cohorts should be conducted to obtain more accurate metabolite findings. In addition, it is necessary to investigate and validate the detailed correlation between protein expression and the related metabolites mentioned in this study to further broaden their therapeutic potential in neurodegenerative diseases such as TBI.

## 5 CONCLUSION

In summary, untargeted LC‐MS/MS metabolomic profiling combined with bioinformation analysis detected global changes in the hippocampus after TBI in the acute phase, which were mainly linked to inflammation, excitotoxicity, oxidative stress, fatty acid beta‐oxidation, and energy requirements. Specifically, a panel of pathway alterations caused significant impairment in taurine and hypotaurine metabolism and arginine and proline metabolism according to KEGG analysis. More importantly, the involvements of taurine and hypotaurine metabolism and arginine and proline metabolism were also determined from a proteomics database analysis in this study. Our findings provide new insight into the relationship between metabolites and proteins for determining the underlying mechanism associated with TBI disease. These novel findings might lead to promising evidence for the development of effective therapeutic strategies for clinical applications in brain damage.

## CONFLICT OF INTEREST

The authors declare no conflict of interest.

## Data Availability

The data that support the findings of this study are available from the corresponding author upon reasonable request.
